# *In Vitro* Reconstitution of SARS-Coronavirus mRNA Cap Methylation

**DOI:** 10.1371/journal.ppat.1000863

**Published:** 2010-04-22

**Authors:** Mickaël Bouvet, Claire Debarnot, Isabelle Imbert, Barbara Selisko, Eric J. Snijder, Bruno Canard, Etienne Decroly

**Affiliations:** 1 Architecture et Fonction des Macromolécules Biologiques, CNRS and Universités d'Aix-Marseille I et II, UMR 6098, ESIL Case 925, Marseille, France; 2 Molecular Virology Laboratory, Department of Medical Microbiology, Center of Infectious Diseases, Leiden University Medical Center, Leiden, The Netherlands; University of California Irvine, United States of America

## Abstract

SARS-coronavirus (SARS-CoV) genome expression depends on the synthesis of a set of mRNAs, which presumably are capped at their 5′ end and direct the synthesis of all viral proteins in the infected cell. Sixteen viral non-structural proteins (nsp1 to nsp16) constitute an unusually large replicase complex, which includes two methyltransferases putatively involved in viral mRNA cap formation. The S-adenosyl-L-methionine (AdoMet)-dependent (guanine-N7)-methyltransferase (N7-MTase) activity was recently attributed to nsp14, whereas nsp16 has been predicted to be the AdoMet-dependent (nucleoside-2′O)-methyltransferase. Here, we have reconstituted complete SARS-CoV mRNA cap methylation *in vitro*. We show that mRNA cap methylation requires a third viral protein, nsp10, which acts as an essential trigger to complete RNA cap-1 formation. The obligate sequence of methylation events is initiated by nsp14, which first methylates capped RNA transcripts to generate cap-0 ^7Me^GpppA-RNAs. The latter are then selectively 2′O-methylated by the 2′O-MTase nsp16 in complex with its activator nsp10 to give rise to cap-1 ^7Me^GpppA_2′OMe_-RNAs. Furthermore, sensitive *in vitro* inhibition assays of both activities show that aurintricarboxylic acid, active in SARS-CoV infected cells, targets both MTases with IC_50_ values in the micromolar range, providing a validated basis for anti-coronavirus drug design.

## Author Summary

In 2003, an emerging coronavirus (CoV) was identified as the etiological agent of severe acute respiratory syndrome (SARS). SARS-CoV replicates and transcribes its large RNA genome using a membrane-bound enzyme complex containing a variety of viral nonstructural proteins. A critical step during RNA synthesis is the addition of a cap structure to the newly produced viral mRNAs, ensuring their efficient translation by host cell ribosomes. Viruses generally acquire their cap structure either from cellular mRNAs (e.g., “cap snatching” of influenza virus) or employ their own capping machinery, as is supposed to be the case for coronaviruses. mRNA caps synthesized by viruses are structurally and functionally undistinguishable from cellular mRNAs caps. In coronaviruses, methylation of mRNA caps seems to be essential, since mutations in viral methyltransferases nsp14 or nsp16 render non-viable virus. We have discovered an unexpected key role for SARS-CoV nsp10, a protein of previously unknown function, within mRNA cap methylation. Nsp10 induces selective 2′O-methylation of guanine-N7 methylated capped RNAs through direct activation of the otherwise inactive nsp16. This finding allows the full reconstitution of the SARS-CoV mRNA cap methylation sequence *in vitro* and opens the way to exploit the mRNA cap methyltransferases as targets for anti-coronavirus drug design.

## Introduction

**Figure 1:**
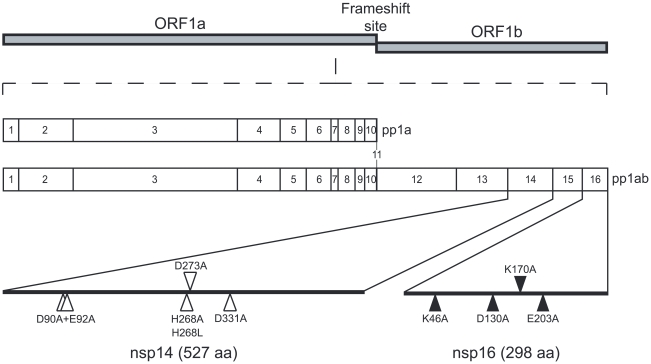
Genomic organization of CoV pp1a/pp1ab and location of the nsp14 and nsp16 mutants. The SARS-CoV genomic RNA is translated in two large polyproteins, pp1a and pp1ab following a -1 ribosomal frame shift. The two polyproteins are then cleaved by viral proteases in order to produce 16 nsps (nsp1 to nsp11 from pp1a and nsp1 to nsp16 from pp1ab). Positions of the point mutants used in this study are indicated. White triangles are used for positions targeting exonuclease motifs of nsp14 and black triangles are used for positions targeting MTase motifs of nsp14 and nsp16 (the putative AdoMet binding site of nsp14 and catalytic tetrad of nsp16).

In 2003, the severe acute respiratory syndrome coronavirus (SARS-CoV), which was likely transmitted from bats, was responsible for a worldwide SARS-outbreak [1]. Coronaviruses belong to the order *Nidovirales* and are characterized by the largest positive-strand RNA ((+) RNA) genomes (around 30,000 nt) known in the virus world. The enzymology of their RNA synthesis is therefore thought to be significantly more complex than that of other RNA virus groups [2], [3], [4]. The 5′-proximal two-thirds of the CoV genome (open reading frames 1a and 1b) are translated into the viral replicase polyproteins pp1a and pp1ab (Figure 1), which give rise to 16 nonstructural proteins (nsps) by co- and post-translational autoproteolytic processing. The 3′-proximal third encodes the viral structural proteins and several so-called accessory proteins, which are expressed from a set of four to nine subgenomic (sg) mRNAs. The latter are transcribed from subgenome-length minus-strand templates, whose production involves a unique mechanism of discontinuous RNA synthesis (reviewed by [5], [6]). To organize their complex RNA synthesis and genome expression, the CoV proteome includes several enzyme activities that are rare or lacking in other (+) RNA virus families (reviewed in [2]). In the years following the 2003 SARS outbreak, bioinformatics, structural biology, (reverse) genetics and biochemical studies have contributed to the in-depth characterization of CoV nsps in general and those of SARS-CoV in particular [7]. Currently documented enzyme activities include two proteinases (in nsp3 and nsp5; [8], [9]), a putative RNA primase (nsp8; [10]), an RNA-dependent RNA polymerase (nsp12; [11], [12]), a helicase/RNA triphosphatase (nsp13; [13], [14]), an exo- and an endoribonuclease (nsp14 and nsp15; [15], [16], and an *S*-adenosyl-L-methionine (AdoMet)-dependent (guanine-N7)-methyltransferase (N7-MTase), which were proposed to play a role in the formation of CoV mRNA caps (nsp14; [17]). Based on comparative sequence analysis, nsp16 presumably encodes an AdoMet-dependent mRNA cap (nucleoside-2′O)-methyltransferase (2′O-MTase) [3], [18], [19]. For SARS-CoV nsp16, however, this enzyme activity has remained elusive thus far, and experimental evidence for its existence has only been obtained for the related feline coronavirus (FCoV) nsp16 [18]. CoV nsps form the viral replication/transcription complex (RTC), which is thought to localize to a network of endoplasmic reticulum-derived, modified membranes in the infected cell [20], [21]. Protein-protein interactions were proposed to be essential for the assembly of the RTC and may therefore also regulate the activities of enzymes involved in viral RNA synthesis.

Although the 5′ ends of SARS-CoV mRNAs have not been characterized yet, they are assumed to carry a cap structure. This assumption is based on the characterisation of genomic and subgenomic mRNAs of the coronavirus murine hepatitis virus (MHV) [22], [23] and the related equine torovirus (EToV or Berne virus), which also belong to the *Coronaviridae* family [23], [24]. The mRNAs of both viruses were concluded to carry a 5′-terminal cap structure. Moreover, in the coronavirus and torovirus genome three enzymes putatively involved in mRNA capping have been identified, although they remain poorly characterised [13], [14], [17], [18], [19]. Cap structures promote initiation of translation and protect mRNAs against exoribonuclease activities [25], [26], [27]. The synthesis of the cap structure in eukaryotes involves three sequential enzymatic activities: (i) an RNA triphosphatase (RTPase) that removes the 5′ γ-phosphate group of the mRNA; (ii) a guanylyltransferase (GTase) which catalyzes the transfer of GMP to the remaining 5′-diphosphate terminus; and (iii) an N7-MTase that methylates the cap guanine at the N7-position, thus producing the so-called “cap-0 structure”, ^7Me^GpppN. Whereas lower eukaryotes, including yeast, employ a cap-0 structure, higher eukaryotes convert cap-0 into cap-1 or cap-2 structures [25], [26], [28] by means of 2′O-MTases, which methylate the ribose 2′O-position of the first and the second nucleotide of the mRNA, respectively. RNA cap methylation is essential since it prevents the pyrophosphorolytic reversal of the guanylyltransfer reaction, and ensures efficient binding to the ribosome [25], [26]. In the case of (+) RNA viruses such as alphaviruses and flaviviruses, mutations in RNA cap methylation genes were shown to be lethal or detrimental to virus replication [29], [30], [31], [32], [33]. For coronaviruses, a functional and genetic analysis performed on MHV temperature sensitive mutants mapping to the N7-MTase domain of CoV nsp14 and in the 2′O-MTase nsp16 indicated that both are involved in positive-strand RNA synthesis by previously formed replicase-transcriptase complexes [11]. The importance of nsp14 and nsp16 for viral RNA synthesis is further supported by data obtained by mutagenesis of MTase catalytic residues in SARS-CoV RNA replicon systems [17], [30].

In the case of coronaviruses, the machinery putatively involved in equipping both genome and subgenomic mRNAs with a cap-1 structure is thought to consist of (i) the multifunctional nsp13, which may contribute the RTPase activity of the helicase domain [13], [34], (ii) a still unknown GTase, (iii) the C-terminal domain of nsp14, which was recently identified as the N7-MTase [17] and (iv) nsp16, the putative 2′O-MTase [3], [17], [18].

Using mammalian and yeast two-hybrid systems as well as pull-down assays, it was shown that SARS-CoV nsp14 and nsp16 specifically interact with nsp10 [35], [36] suggesting that nsp10 may play a role in the viral capping pathway. The crystal structure of nsp10, a small RNA-binding protein that contains two zinc fingers, was recently solved [37], [38], but its role and mode of action in the viral replicative cycle remains elusive. In view of the phenotype of some mouse hepatitis virus (MHV) mutants, a role in viral RNA synthesis was postulated [11], [39], but other studies implicated nsp10 in replicase polyprotein processing [40]. SARS-CoV nsp10 was also shown to bind single- and double-strand RNA and DNA with low affinity and without obvious sequence specificity [37].

In this study, we report the discovery of a function for SARS-CoV nsp10 as an essential factor to trigger full nsp16 2′O-MTase activity. We deciphered the RNA cap methylation sequence where the guanine-N7-methylation by nsp14 necessarily precedes the 2′O-methylation by the nsp10/nsp16 pair. The SARS-CoV nsp10/nsp14/nsp16 trio constitutes an attractive target package for antiviral drug discovery and design; and indeed nsp14 and nsp16 seem to play an important role in viral replication [11], [17], [30]. Accordingly, we set up sensitive inhibition tests for both activities, validated by low IC_50_ values of known AdoMet-dependent MTase inhibitors. Moreover, we show that aurintricarboxylic acid (ATA), which was shown to inhibit SARS-CoV replication [41], targets both MTases indeed.

## Results

### SARS-CoV nsp14 is active as AdoMet-dependent N7-MTase on short capped RNA substrates whereas the nsp16 2′O-MTase requires nsp10 as co-factor

**Figure 2:**
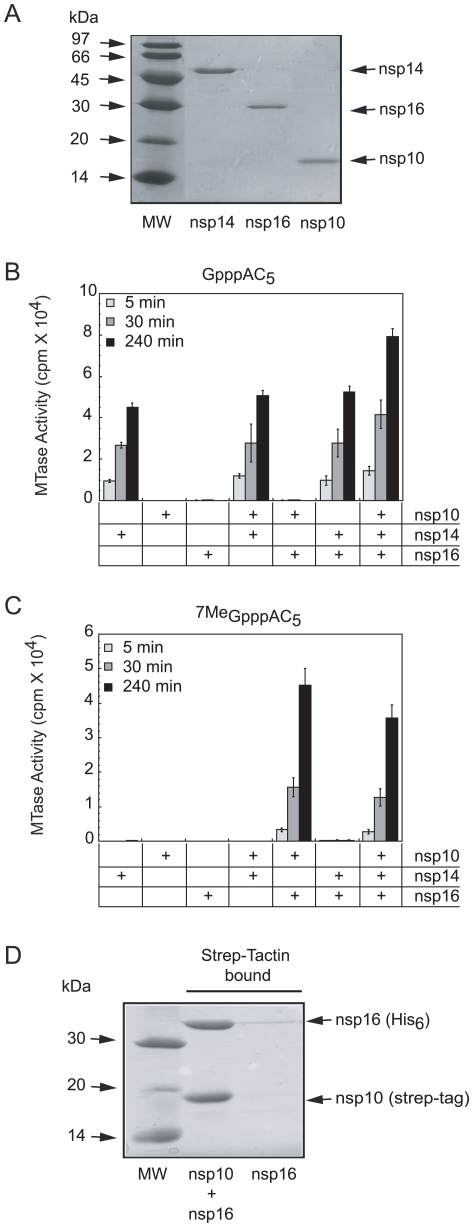
SARS-CoV proteins nsp14, nsp16 and nsp10 purification, AdoMet-dependent MTase activity on short capped RNA and complex formation of nsp16/nsp10. Panel **A**: The SARS-CoV proteins nsp14, nsp16 and nsp10, purified by affinity and size exclusion chromatography (as described in Materials and Methods) were separated by SDS-PAGE (14%) and visualized by Coomassie blue staining. Lane 1 corresponds to the molecular size markers, lanes 2 to 4 to nsp14, nsp16, and nsp10, respectively. Panel **B and C**: AdoMet-dependent MTase assays performed on short capped RNA substrates. The different purified proteins (nsp10: 1200 nM, nsp14: 50 nM and nsp16: 200 nM) were incubated with GpppAC_5_ and ^7Me^GpppAC_5_ RNA oligonucleotides in presence of [^3^H]-AdoMet as described in Materials and Methods. The methyl transfer to the capped RNA substrate was determined after 5-, 30-, and 240-min incubation by using a filter-binding assay (see Materials and Methods). Panel **D**: SARS-CoV His_6_-nsp16 protein co-expressed with strep-tag-nsp10 and His_6_-nsp16 expressed alone were incubated with Strep-Tactin sepharose. Strep-Tactin-bound protein was eluted with D-desthiobiotin and analysed by SDS-PAGE and Coomassie blue staining. Lane 1 corresponds to the molecular size markers, lane 2 to strep-tag-nsp10 co-expressed with His_6_-nsp16 and lane 3 to His_6_-nsp16 alone.

Unlike flaviviruses, which use a single active site in the NS5 protein for both N7- and 2′O-MTase activities [32], [42], coronaviruses presumably encode two separate MTases that catalyze the last two steps in the formation of a methylated RNA-cap structure. SARS-CoV nsp14 has been shown to be an RNA-cap N7-MTase [17]. Sequence motifs that are canonical in 2′O-MTases were identified in nsp16 [3], [19], but the experimental verification of the MTase activity has not been reported, in contrast to FCoV nsp16, for which a rather low activity could be demonstrated [18]. SARS-CoV nsp10 was previously shown to interact with both nsp14 and nsp16 [35], [36], suggesting its involvement in RNA capping and/or methylation. Consequently, we cloned and expressed both nsp10 and nsp16 in *E. coli* and purified both recombinant proteins, using their N-terminal His_6_-tag, by metal affinity chromatography. Nsp14 was expressed as a fusion protein with an intein tag at its C-terminus. The nsp14-intein product was bound to a chitin affinity column and the untagged protein was eluted after removal of the tag by DTT treatment. All three proteins were further purified by size exclusion chromatography. Upon SDS-PAGE, the purified proteins migrated as single bands corresponding to their expected molecular masses (nsp14: 57 kDa; His_6_-nsp16: 35 kDa, and His_6_-nsp10: 15 kDa) (Figure 2A). The identity of the recombinant proteins was confirmed by trypsin digestion and mass spectrometry ((MALDI-TOF), data not shown).

Using the purified recombinant proteins, we first conducted *in vitro* MTase assays on short capped RNA substrates methylated or not at the N7-position of the guanine cap (^7Me^GpppAC_5_ and GpppAC_5_). We used all possible combinations of the three proteins (nsp10, nsp14, and nsp16) and incubated them with the substrate in the presence of the tritiated methyl donor [^3^H]-AdoMet. The extent of [^3^H]-CH_3_ transfer was quantified after reaction times of 5, 30, and 240 min by using a DEAE filter-binding assay (see Materials and Methods). Figure 2B shows that nsp14 methylated GpppAC_5_ in a time-dependent manner whereas neither nsp16 nor nsp10 alone did. Apparently, the activity of nsp14 was barely influenced by the presence of nsp10 or nsp16. In addition, we observed that nsp14 did not methylate ^7Me^GpppAC_5_ (Figure 2C) suggesting that nsp14 methylates only the N7-position of the cap structure. In contrast to nsp14, nsp16 catalyzed methyltransfer to neither GpppAC_5_ nor to ^7Me^GpppAC_5_ under these reaction conditions. Surprisingly, when nsp16 activity assays were supplemented with nsp10, robust methylation of ^7Me^GpppAC_5_ was observed (Figure 2C), but not of GpppAC_5_ (Figure 2B). In control reactions, containing either nsp10 alone or nsp10 supplemented with nsp14 no ^7Me^GpppAC_5_-specific MTase activity was detected (Figure 2C). When the GpppAC_5_ substrate was incubated with a combination of nsp10, nsp14, and nsp16 (Figure 2B)_,_ the level of substrate methylation was enhanced compared to reactions performed with nsp14 alone. After overnight incubation of GpppAC_5_ with the three proteins, the methyl incorporation reached a plateau and the incorporation level was twice higher than after a reaction in the presence of nsp14 alone (not shown). In contrast, no significant difference was observed between the methylation level reached after incubation of the ^7Me^GpppAC_5_ substrate with either all three proteins or the nsp16-nsp10 pair only (Figure 2C). Taken together, these results suggest that, (i) SARS-CoV nsp14 methylates GpppAC_5_ at the N7-position of the cap guanine and indeed acts as an N7-MTase on these substrates, (ii) nsp16 acts as an nsp10-dependent 2′O-MTase on ^7Me^GpppAC_5_, (iii) the 2′O-MTase activity of nsp16-nsp10 requires the presence of a cap structure already methylated at its N7-position and (iv) nsp14 and the nsp16-nsp10 pair can perform sequential double methylation of GpppAC_5_, presumably at the N7- and 2′O-positions.

To determine how nsp10 stimulated nsp16 MTase activity, we co-expressed in *E. coli* an N-terminally Strep-tagged nsp10 and a His_6_-tagged nsp16. The bacterial cell lysate containing these proteins was incubated with Strep-Tactin beads (see Materials and Methods), in order to bind the tagged nsp10. After extensive washing, the proteins bound to the beads were analysed using SDS-PAGE. Figure 2D indicates that nsp16 remained associated with nsp10, whereas nsp16 alone was unable to bind to the beads. These data suggest that nsp10 can stimulate the MTase activity of nsp16 by direct association resulting in the formation of a nsp10/nsp16 complex. When the intensities of the bands corresponding to nsp10 and nsp16 were quantified, a ratio of nsp10 to nsp16 of 1.1 was obtained. Correcting for the respective molecular masses, and assuming that they bind Coomassie blue dye with the same affinity, this yields a nsp10 to nsp16 ratio of about 2.3. This suggests that the complex does not contain a large molar excess of nsp10, as one might have expected due to the fact that nsp10 seems to form dodecamers under certain conditions [38].

### mRNA cap N7- and 2′O-site specific methylation by SARS-CoV nsp14 and nsp10/nsp16

**Figure 3:**
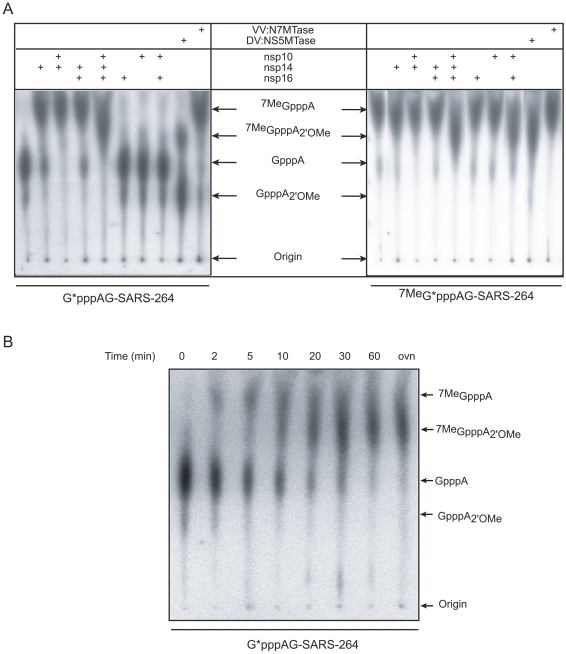
AdoMet-dependent MTase assays of nsp14 and nsp16/nsp10 on long, virus-specific, capped RNA substrates. Panel **A**: Capped RNAs corresponding to the first 264 nucleotides of the SARS-CoV genome were incubated with SARS-CoV proteins (nsp10: 1.2 µM, nsp14: 50 nM and nsp16: 200 nM). Labeled substrates G*pppAG-264 or ^7Me^G*pppAG-264 RNA (the asterisk indicates the labeled phosphate) were incubated alone or in presence of the indicated proteins, digested by nuclease P1 and analyzed by TLC. The origins and the positions of standards GpppA, ^7Me^GpppA, ^7Me^GpppA_2'__OMe_ and GpppA_2'__OMe_ (see Materials and Methods) are indicated by black arrows. VV:N7-MTase stands for vaccinia virus N7-MTase and DV:NS5MTase for dengue virus MTase domain of protein NS5, a bi-functional N7- and 2′O-MTase, which were used as positive controls. Panel **B**: Time course analysis of the N7- and 2′O-methylation by nsp14 and nsp16/nsp10. Labeled G*pppAG-SARS-264 RNA was incubated with a mixture of nsp10 (1.2 µM), nsp14 (50 nM), and nsp16 (200 nM). Methylation of the cap structure was followed during 60 min. The final point (overnight  =  ovn) corresponds to 20 h. As in panel A, TLC analysis of nuclease P1-resistant cap structures is shown. The positions of the origin of migration and of GpppA, ^7Me^GpppA, ^7Me^GpppA_2'__OMe_ and GpppA_2'__OMe_ cap analog standards are indicated.

In order to test MTase activities of nsp14 and nsp10/nsp16 on virus-specific capped RNA substrates, we synthesized a 5′-triphosphate-carrying RNA corresponding to the first 264 nucleotides of the SARS-CoV genome using the T7 RNA polymerase. Since the canonical T7 promoter inefficiently directs transcription of RNA beginning with an A, as is required to make transcripts resembling the 5′ end of coronavirus RNAs, we used the T7 class II φ2.5 promoter [43]. Additionally, we introduced a U→G substitution in the 2^nd^ position of the RNA to increase the *in vitro* transcription efficiency (data not shown). The RNA was capped with [α-^32^P]-GTP using the vaccinia virus (VV) capping enzyme (containing RTPase, GTase and N7-MTase activities, see Materials and Methods) in the presence or absence of the methyl donor AdoMet. The substrates GpppAG-SARS-264 and ^7Me^GpppAG-SARS-264 were then incubated with various combinations of nsp14, nsp16, and nsp10. Reaction products were digested by nuclease P1 in order to release the RNA cap structure. Radiolabeled cap molecules were subsequently separated on TLC plates and visualized using autoradiography. The comparison with commercially available and in-house synthesized cap analogs allowed the identification of the methylation position of the cap structure. Figure 3A shows that the cap structure released after nuclease P1 digestion of substrates GpppAG-SARS-264 and ^7Me^GpppAG-SARS-264 RNA co-migrated, as expected, with GpppA and ^7Me^GpppA cap analogs, respectively. In the presence of nsp14, or the VV:N7-MTase positive control, the GpppA cap structure present at the 5′ end of the RNA was converted into ^7Me^GpppA (left panel of Figure 3A). We also observed that the methylation of the N7-position induced by nsp14 was weakly stimulated in the presence of nsp10, but was not influenced by the presence of nsp16. Indeed, nsp14 converts 83% of the substrate into the ^7Me^GpppA product, whereas in the presence of nsp10 97% of the substrate was converted, as judged by autoradiography analysis. Nsp10 or nsp16 alone did not show any MTase activity. When all three proteins are present, the substrate is fully methylated at the N7- and 2′O-positions of the cap, as judged by the comparison with products generated by the bifunctional N7- and 2′O-MTase domain of dengue virus protein NS5 (DV:NS5MTase), which was used as a positive control [32], [42]. The right panel of Figure 3A shows that incubation of ^7Me^GpppAG-SARS-264 RNA, with nsp14, nsp16 or nsp10 alone did not result in 2′O-methylation of the ^7Me^GpppA structure. The same was true when nsp14/nsp10 or nsp14/nsp16 combinations were tested. In contrast, 2′O-methylation of the cap structure of ^7Me^GpppAG-SARS-264 occurred upon incubation with nsp10/nsp16, and also when all three proteins were used together. We therefore conclude that capped RNA corresponding to the first 264 nucleotides of the SARS-CoV genome represents a *bona fide* substrate to follow the RNA cap MTase activities of SARS-CoV nsp14 and nsp10/nsp16. Moreover, the TLC analysis allowed us to demonstrate that nsp14 indeed specifically methylates RNA cap structures at the N7-position and that nsp10/nsp16 methylates capped RNA at the 2′O-position of the first nucleotide after the N7-methylated cap. As also observed when using short substrates, nsp10/nsp16 could only methylate ^7Me^GpppAG-SARS-264 and not GpppAG-SARS-264, suggesting that N7-methylation by nsp14 must precede 2′O-methylation by nsp10/nsp16. We conclude that nsp14 exhibits N7-MTase activity in the absence of nsp10, whereas the latter is an absolute requirement for nsp16-mediated 2′O-methylation of the cap structure. Nsp10, which was previously shown to interact with both nsp14 and nsp16 [35], [36], modestly stimulates the nsp14-mediated cap N7-MTase activity (Figure 3A and S1B; 10 to 15% increase of activity at a broad optimum around a 4-fold molar excess).

In order to directly monitor the order of SARS-CoV RNA-cap methylation, we performed a time-course experiment using the GpppAG-SARS-264 substrate in conjunction with nsp10, nsp14, and nsp16. The results, shown in Figure 3B, indicate that methylation of the substrate indeed starts at the N7-position. Subsequently, the ^7Me^GpppA cap-0 structure is converted to an ^7Me^GpppA_2'__OMe_ cap-1 structure. A GpppA_2'__OMe_ structure was never observed in this assay, not even when using larger amounts of nsp10/nsp16 or nsp16 (data not shown), in agreement with the data presented in Figures 2B and 3A that show that GpppAC_5_ and GpppAG-SARS-264 substrates are not methylated by nsp10/nsp16. Thus, the N7-methylation of the SARS-CoV cap structure by nsp14 is a pre-requisite for its recognition by the nsp10/nsp16 pair, which then converts the cap-0 into a cap-1 structure by 2′O-methylation.

### The nsp14 D_331_ residue is essential for N7-methylation whereas catalytic residues of the N-terminal exonuclease domain are not

**Figure 4:**
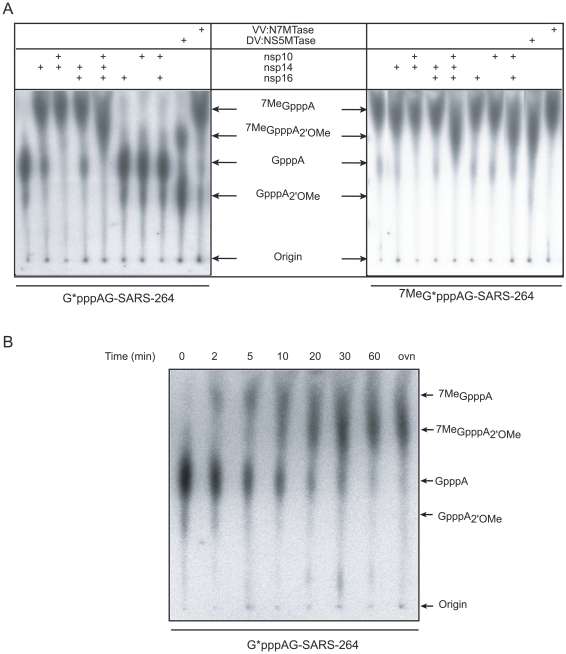
Alanine mutagenesis of nsp14 and nsp16 proteins. Panel **A**: Residues of the nsp14 exoribonuclease and MTase catalytic sites were mutated to alanine as indicated in Materials and Methods. Equal amounts (50 nM) of each nsp14 mutant were incubated with GpppAC_5_ in the presence of [^3^H]-AdoMet. Methyl transfer to the RNA substrate was measured after 30 min by using a filter-binding assay (upper panel). The N7-MTase activity of the wt control protein was arbitrarily set to 100%. The bar graph presents the results of 3 independent experiments. The purified His_6_-tagged proteins analyzed by SDS-PAGE (14%) are shown in the lower panel. Panel **B**: Each residue of the putative catalytic tetrad K_46_-D_130_-K_170_-E_203_ of nsp16 was mutated to alanine. Equal amounts of the different nsp16 mutants (200 nM) were incubated with ^7Me^GpppAC_5_ in the presence of [^3^H]-AdoMet and nsp10 (1.2 µM). The methyl transfer to the RNA substrate was measured after 30 min by using a filter-binding assay. The 2′O-MTase activity of the wt protein in the presence of nsp10 was arbitrarily set to 100%. The bar graph represents the mean of 3 independent experiments. The purified His_6_-tagged proteins analyzed by SDS-PAGE (14%) are shown in the lower panel.

The recent identification of the C-terminal domain of nsp14 as an N7-MTase [17] revealed that this replicase subunit is a multifunctional protein, since it also carries an exoribonuclease activity embedded in its N-terminal domain [16]. The interplay between these two functionalities was analyzed using mutagenesis experiments. We mutated conserved residues in both the MTase and the exoribonuclease domain to evaluate the possible interplay or long-range regulation of both activities. The conserved residue D_331_, which is presumably involved in AdoMet-binding, was mutated to alanine. In the exoribonuclease domain, we replaced conserved residues from exonuclease motifs I (D_90_XE_92_), II (D_243_) and III (D_273_ and H_268_) of the DE(A/D)D nuclease superfamily. All the His-tagged nsp14 mutant proteins could be expressed, except the D243A mutant, which was barely soluble. Figure 4A shows that they migrated at a molecular mass similar to that of wt nsp14 upon SDS-PAGE. We next analyzed their N7-MTase activity on GpppAC_5_ using [^3^H]-AdoMet as methyl donor. The results show that the D331A point mutation completely abolished nsp14 N7-MTase activity. This is in agreement with the hypothesis [17] that the MTase domain is located in the C-terminal half of nsp14 protein and that the conserved residue D_331_ is important for N7-MTase function. In contrast, the mutations in the exonuclease domain did not significantly interfere with nsp14 MTase activity, excepted in the case of the motif I-double mutant (D_90_XE_92_) which displayed attenuated N7-MTase activity (∼2-fold). This observation is in agreement with the fact that a N-terminal truncation of 90 amino acids of the nsp14 exoribonuclease domain abolished the N7-MTase activity in a yeast trans-complementation assay [17]. Thus an altered N-terminus of the exoribonuclease domain may still interfere with the MTase activity to a certain extent.

### Identification of K_46_-D_130_-K_170_-E_203_ as a catalytic tetrad in SARS-CoV nsp16

In order to ascertain that nsp16 supports the 2′O-MTase activity and not the nsp10 protein, we engineered and characterized a set of nsp16 point mutations. We mutated the conserved residues K_46_-D_130_-K_170_-E_203,_ which form the canonical catalytic tetrad of mRNA cap 2′O-MTases [42], [44]. The putative catalytic residues of SARS-CoV nsp16 were identified using sequence alignment with the homologous FCoV nsp16 2′O-MTase and other family members [18]. Three of the four alanine point mutants could be expressed as efficiently as wt nsp16, allowing their purification to homogeneity using a single-step of affinity chromatography (see Materials and Methods). Still, smaller amounts of the fourth mutant (K46A) could also be produced and purified. We obtained sufficient soluble protein to perform MTase assays, although protein yield and purity were lower than for the other mutants (Figure 4B). For all mutant proteins, the 2′O-MTase activity was tested on ^7Me^GpppAC_5_ and compared to that of the wt nsp16/nsp10 control pair. The 2′O-MTase activity was indeed completely abolished by any single mutation of the putative K_46_-D_130_-K_170_-E_203_ catalytic tetrad residues of nsp16. This result demonstrated that although nsp10 stimulates the 2′O-MTase activity by a yet unknown mechanism, the catalytic activity itself resides in nsp16.

### Inhibition of MTase activities of SARS-CoV nsp14 and nsp16/nsp10

Viral MTases exhibit many original features relative to their host cell MTase counterparts, and are increasingly explored as putative targets for the development of antivirals [45]. In order to set up sensitive N7- and 2′O-MTase inhibition tests, we determined more precisely the conditions to measure optimum MTase activity for nsp14 and nsp16/nsp10 using their respective substrates GpppAC_5_ and ^7Me^GpppAC_5_ (see Text S1). We thus defined the following standard assay conditions: the N7-MTase activity of nsp14 was measured in presence of 40 mM Tris-HCl, pH 8.0 and 5 mM DTT. For the 2′O-MTase assays, the same buffer was used together with 1 mM of MgCl_2_. As Figure S1B illustrates, nsp10 stimulates nsp16 2′O-MTase activity in a dose-dependent manner. We used a 6-fold molar excess of nsp10 over nsp16 corresponding to ≈75% of the maximal stimulation that could be achieved.

Inhibition was tested for two AdoMet analogs with documented mRNA cap MTase inhibition properties: AdoHcy (S-adenosyl-l-homocysteine), the co-product of methyl transfer, and sinefungin [18], [46], [47], [48], [49]. We also used compounds known to target other AdoMet-dependent MTases, such as SIBA, 3-deaza-adenosine [50] and MTA [51]. Based on their adenosine or guanosine-containing structures, adenosine- and AdoMet-analogs 2′,3′,5′-tri-O-acetyl-adenosine and S-5′-adenosyl-L-cysteine were tested as well as GTP, ^7Me^GTP, GTP- or cap-analogs (ribavirin and its triphosphate as well as EICAR-triphosphate, GpppA and ^7Me^GpppA). Finally, we included two inhibitors of flavivirus mRNA cap MTase activites: aurintricarboxylic acid (ATA), which is expected to bind to the MTase active site [52], and a substituted adamantane compound supposedly binding to the AdoMet-binding site [53]. Interestingly, ATA has been shown recently to inhibit SARS-CoV replication by an unknown mechanism of action [41].

**Figure 5:**
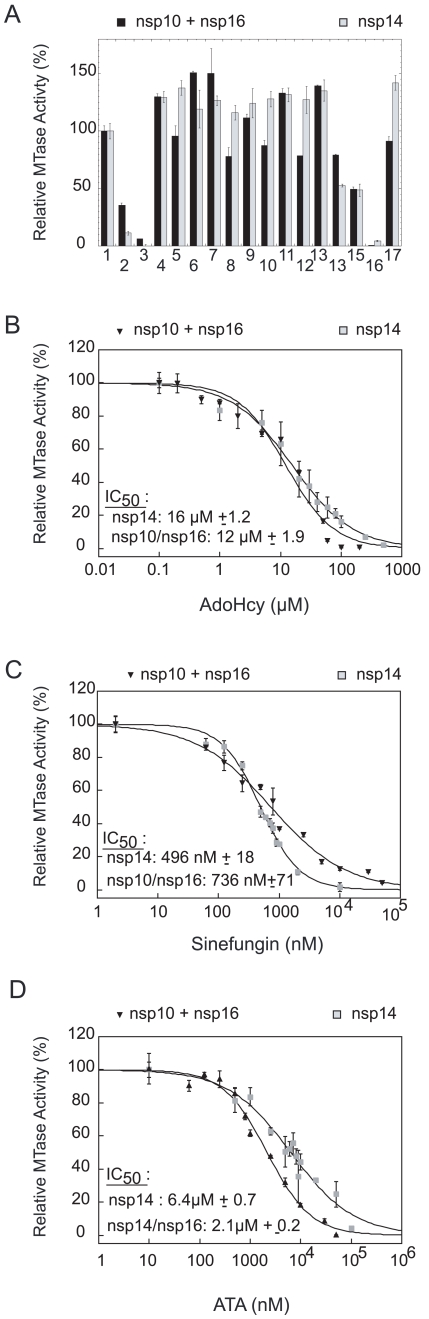
Inhibition of the nsp14 and nsp16/nsp10 MTase activities. Nsp14 (50 nM) and nsp16/nsp10 (200 nM/1.2 µM) were incubated with GpppAC_5_ (in grey) and ^7Me^GpppAC_5_ (in black), respectively, in order to measure the methyl transfer to the RNA substrates by filter-binding assay (see Materials and Methods). Panel **A**: Methyl transfer was measured at a final concentration of 100 µM of each inhibitor candidate. The outcome of the control reaction in absence of inhibitor and at 5% of DMSO was set to 100%. The mean value of three independent experiments is given. 1: control, 2: AdoHcy, 3: sinefungin, 4: SIBA (5′-S-isobutylthio-5′-deoxyadenosine), 5: 3-deaza-adenosine, 6: MTA (5′-deoxy-5′-methylthio-adenosine), 7: 2′,3′,5′-tri-O-acetyl-adenosine, 8: S-5′-adenosyl-L-cysteine, 9: GTP, 10: ^7Me^GTP, 11: ribavirin, 12: ribavirin-triphosphate, 13: EICAR-triphosphate, 14: GpppA, 15: ^7Me^GpppA, 16: ATA, 17: adamantane-analog (N-({[3-(4-methylphenyl)-1-adamantyl]amino}carbonyl)phenylalanine). Panels **B, C and D**: Dose-response curves and IC_50_ values of inhibitors AdoHcy, sinefungin and ATA, respectively. The results of three independent experiments are given. Standard deviations are shown for concentrations that were tested three times. IC_50_ values were calculated as described in Materials and Methods.

Nsp14 and nsp16/nsp10 were first incubated with 100 µM of each candidate inhibitor in the presence of [^3^H]-AdoMet. N7- and 2′O-MTase activities were determined by quantification of methyl transfer to the GpppAC_5_ and ^7Me^GpppAC_5_ RNA substrates, respectively. As shown in Figure 5A, 10 out of 16 tested molecules barely inhibited the SARS-CoV MTases. Cap analogs (GpppA and ^7Me^GpppA) showed limited (50%) inhibition capacity on both SARS-CoV MTases. In contrast, we observed that AdoHcy, sinefungin and ATA efficiently inhibited both enzymes at 100 µM. The IC_50_ values of AdoHcy were 16 and 12 µM for the N7- and 2′O-MTase activities, respectively (Figure 5B) ten-fold higher than Ki values reported for VV:N7- and 2′O-MTases (1 and 0.5 µM, respectively, [47]). Sinefungin, a potent inhibitor of VV:N7- and 2′O-MTases with reported IC_50_ values of 12.0 and 39.5 nM, respectively [46], showed the most potent inhibition profile on nsp14 and nsp10/nsp16 with IC_50_ values of 496 nM and 736 nM, respectively (Figure 5C). These values are similar to the IC_50_ value reported for the inhibition of the 2′O-MTase activity of DV:NS5MTase (420 nM [49] and 630 nM [48]). The obtained IC_50_ values of ATA for SARS-CoV nsp14 and nsp10/nsp16 were 6.4 µM and 2.1 µM, respectively (Figure 5D). These results demonstrate that sensitive assays are now available to discover and characterize inhibitors of the SARS-CoV N7- and 2′O-MTases rendering low IC_50_ values of known AdoMet-dependent MTase inhibitors like AdoHcy and sinefungin. Moreover we have shown that nsp14 and nsp16 MTases are two putative targets of ATA, that was shown to inhibit SARS-CoV replication in infected cells [41].

## Discussion

Enzymatic activities postulated to be involved in the SARS-CoV RNA capping pathway were previously documented for the ORF1b-encoded replicase subunits nsp13 (RNA 5′- triphosphatase/helicase [13]) and nsp14 (N7-MTase [17]). Thus far, the predicted 2′O-MTase activity of nsp16 [3], [19] could only be verified for FCoV nsp16 [18]. Surprisingly, SARS-CoV nsp16 2′O-MTase failed to exhibit activity under a wide range of experimental conditions, including those used for FCoV nsp16 (not shown). In this study, we have characterized the MTase activities of both SARS-CoV nsp14 and nsp16, and in particular established that the *in vitro* activity of SARS-CoV nsp16 critically depends on the presence of nsp10. The latter had no known role or function, but was previously shown to interact with both MTase proteins nsp14 and nsp16 [35], [36].

Here, we show that the nsp14 AdoMet-dependent MTase activity can methylate GpppAC_5_ RNA, but not a^ 7Me^GpppAC_5_ substrate, indicating that nsp14 specifically targets the N7-position of the guanine residue in the cap structure. This was verified using a substrate mimicking the capped 5′ end of the SARS-CoV genome. Nuclease P1 enzymatic digestion and TLC analysis confirmed the position of methylation by nsp14; and mutagenesis of a predicted AdoMet binding site residue abolished N7-MTase activity. We therefore conclude that nsp14 alone can act as an AdoMet-dependent MTase that specifically targets the N7-position of the cap structure, thus converting GpppRNA into ^7Me^GpppRNA. These results confirm and extend the recently described observations on the cap N7-MTase activity of SARS-CoV nsp14 *in vitro* and in a yeast-based complementation system [17].

In contrast to nsp14, bacterially expressed SARS-CoV nsp16 is less stable, reluctant to crystallization (not shown), and inactive on ^7Me^GpppRNA and GpppRNA in our *in vitro* assays. We report here that SARS-CoV nsp16 forms a complex with nsp10 that is endowed with robust and long-lived MTase activity. In contrast, FCoV nsp16 by itself was shown to possess 2′O-MTase activity under similar reaction conditions, but at much higher enzyme concentration (SARS-CoV: 200 nM; FCoV: 3 µM [18]). This suggests that FCoV nsp16 might also need FCoV nsp10 for its proper activation. As in the case of FCoV nsp16, SARS-CoV nsp16 in the presence of nsp10, specifically methylates capped RNAs carrying a methyl group at the N7-guanine position, allowing the conversion of cap-0 into cap-1 structures. Using ^7Me^GpppAG-RNA corresponding to the 5′ end of the SARS-CoV genome, we have confirmed that nsp10/nsp16 catalyzes the transfer of a methyl group from the AdoMet donor to the 2′O-position of the first nucleotide after the cap guanosine. Finally, the intrinsic nsp16 2′O-MTase activity was corroborated by mutagenesis of its predicted, canonical catalytic tetrad K_46_-D_130_-K_170_-E_203_ [3], [18], [19].

### The interplay between nsp10 and the nsp16 MTase

The nsp10 protein was previously proposed to play a role in viral RNA synthesis [11], [39], [40] and replicase polyprotein processing [40] on the basis of the analysis of MHV nsp10 mutants. In this work, we propose a new function for nsp10 as a regulator of an enzyme involved in the methylation of cap structures. Our observation seems not directly related to the phenotype previously described for nsp10 mutants [11], [39], [40]. Nevertheless, RNA cap methylation defects should limit RNA stability and may therefore contribute to a decrease in viral RNA synthesis observed in MHV mutants [11], [39], [40]. Here, we show that nsp10 itself is catalytically inert in methylation reactions (Figures 2, and 3) and that it forms a complex with nsp16 (Figure 2C). Interestingly, whereas at least a 10-fold molar excess of nsp10 is required for maximal stimulation of nsp16 (Figure S1B), quantification of the protein bands of nsp10 and nsp16 in the purified complex indicates a maximum ratio of 2.3 (Figure 2D). We assume that, under conditions of maximal stimulation, nearly all nsp16 molecules are associated with one or two nsp10 molecules. Considering that the reaction mixture at 50% stimulation contained 200 nM of nsp16 and around 400 nM of nsp10 (Figure S1B), the dissociation constant of the nsp10/nsp16 complex can roughly be estimated to be in the order of 400 nM for a 1∶1 complex or 200 nM for a 2∶1 complex. This is in agreement with a K_d_ of 250 nM determined by plasmon surface resonance analysis (Lecine P. personal communication).

What could be the mechanism of nsp16 activation by nsp10? Nsp10 may increase the stability of nsp16, stabilize the nsp16 RNA binding groove, contribute to RNA substrate binding and/or allosterically regulate its substrate affinity and activity. Similar activation of an MTase involved in the capping pathway was previously reported for the VV capping enzyme [54]. The catalytic efficiency of the N7-MTase domain of the VV:D1 protein was 370-fold stimulated by addition of an equimolar concentration of the small VV:D12 protein, which does not contain any catalytic residues [55]. Activation is achieved through increase of substrate and co-substrate affinity as well as of the turn over number. At the same time, VV:D12 exerts a stabilizing effect on VV:D1 [55]. The determination of the crystal structure of the protein complex VV:D1/D12 [56] revealed that the VV:D12 protein is structurally homologous to the cap 2′O-MTase of reovirus, with a truncation of the AdoMet binding domain. The SARS-CoV nsp10 crystal structure did not reveal any similarity to an MTase fold nor to any protein in the Protein Data Bank (PDB) [37], [38], but the activating effect of nsp10 on nsp16 might also be exerted on different levels via allosteric activation by increasing substrate affinity and/or turn over number, and/or by stabilization of nsp16.

The presence of two Zn fingers is a major structural feature of nsp10 that is likely related to its biological functions, since Zn fingers typically function as interaction modules binding to proteins, nucleic acid and small molecules [57]. Interestingly, several MTases have previously been shown to be regulated through specific interactions with Zn finger domains [58], [59]. Since the first Zn finger of nsp10 lies in a positively charged surface patch, it might be involved in the low affinity interaction of nsp10 with single- and double-strand RNA and DNA [37]. The affinity of nsp10 for single–stranded RNA appears too weak to explain a direct role in RNA recruitment for nsp16 [36]. We expect that SARS-CoV nsp16 contains a specific binding site for a cap-0 structure followed by a small stretch of 3 to 4 nucleotides as predicted for FCoV nsp16 from enzymatic assays [18]. The formation of the complex nsp10/nsp16 might provide a longer substrate-binding site and in that way enhance affinity of nsp16 for its RNA substrate. Nevertheless, given the fact that the full activation effect by nsp10 is also seen when short substrates containing the cap and 5 nucleotides are used, we surmise that extension of the RNA binding site is of minor importance. Further work is needed in order to understand the molecular basis of nsp16 activation by nsp10.

### Coronavirus mRNA cap methylation

There are indications that CoV genomic RNAs and subgenomic RNAs carry a 5′-terminal cap-1 structure (see Introduction) and three of four putative cap-forming enzyme functions required to produce this structure have now been identified for SARS-CoV (nsp13, nsp14, and nsp16) [3], [17], [18]. The CoV cap structure methylation seems to follow the “classic” sequence of N7-methylation preceding the 2′O-methylation. The modular structure of two separate single-domain enzymes corresponds to the scenario in metazoan and plants [25], [26]. It contrasts to dsRNA reoviruses where one multi-domain protein contains two MTase domains [60] and to flaviviruses and negative-strand RNA ((-) RNA) vesiculoviruses where both MTase activites reside in a single domain of larger proteins and use a single active site [32], [61]. A characteristic feature of CoV MTases is that nsp14 recognizes non-methylated RNA cap exclusively, and nsp10/nsp16 recognizes N7-methylated RNA cap exclusively. In contrast, bi-functional MTases recognize both non-methylated and methylated cap structures with equal affinity [48], [62], [63]. Interestingly, the flaviviral N7-MTase activity is regulated by specific 5′-proximal viral RNA secondary structures and both N7- and 2′O-MTase activities seem to require in particular the terminal dinucleotide AG [32], [64]. Since the SARS-CoV nsp14 N7-MTase activity can complement N7-MTase defects in yeast, [17], it suggests that specific sequences and/or RNA structures are not required for this activity. This was confirmed in our *in vitro* assays, where both N7- and 2′O-methylation was observed using small GpppAC_5_ RNA substrates that do not correspond to the natural sequence present at the 5′ end of CoV mRNAs. Nevertheless, the CoV capping machinery is likely to act specifically on viral mRNA substrates, which present a common 5′-terminal leader sequence (72 nucleotides long in the case of SARS-CoV [4]). The mechanism to achieve this selectivity may depend on the GTase reaction or on the fine regulation of capping enzymes by protein-protein interactions within the replication and transcription complex. The regulation of the 2′O-MTase activity of nsp16 by the small nsp10 protein is clearly an original feature of CoV mRNA cap methylation.

### Coronavirus MTases as potential targets for antiviral inhibitors

Virally encoded RNA cap N7- and 2′O-MTase activities have been identified in various virus families, such as dsDNA poxviruses [65], [66], dsRNA reoviruses [67], [68], (-) RNA viruses such as vesicular stomatitis virus [61], and (+) RNA viruses like flaviviruses [33], [42], [69]. For many of them, including coronaviruses [17], [30], it has been shown that mutations abolishing the N7-MTase activity have a clear detrimental effect on replication [32], whereas 2′O-MTase knockouts exerted more moderate effects [32], [33]. These observations suggest that compounds specifically inhibiting cap MTases could be potent antiviral agents. Although some viral MTase inhibitors have also been reported to inhibit mammalian MTases [70], [71], sinefungin or other AdoMet analogs might have higher specificity towards viral MTases. Accordingly, it has been shown that sinefungin inhibits fungal mRNA cap N7-MTases with 5 to 10 times more potency than the human isoform [71].

Here, we report assays using GpppAC_5_ and ^7Me^GpppAC_5_ substrates that constitute sensitive screening tests for the identification and characterization of inhibitors of the N7- and 2′O-MTase activities, respectively, of SARS-CoV. We confirmed this by obtaining low IC_50_ values of known AdoMet-dependent MTase inhibitors AdoHcy and sinefungin. Furthermore, we found that ATA, a compound previously reported as a putative blocker of the catalytic site of NS5MTase of flaviviruses [52], and of SARS-CoV replication in infected cells [41], inhibited both SARS-CoV MTase activities with IC_50_ values of 2.1 and 6.4 µM, respectively. Thus, we propose that nsp14 and nsp16/nsp10 are two of the SARS-CoV targets of ATA leading, or at least contributing, to the inhibition of SARS-CoV replication.

In conclusion, our results identify and characterize the main viral protein players of SARS-CoV mRNA cap methylation. Its specificity and mechanistic originality remain unparalleled thus far and should open new avenues to investigate viral RNA capping, a field that is increasingly permeable to drug design projects.

## Materials and Methods

### Reagents

AdoMet and cap analogs GpppA and ^7Me^GpppA were purchased from New England BioLabs. The compounds tested as MTase inhibitors were purchased from the following providers: Sigma-Aldrich: AdoHcy (adenosine-homocysteine), GTP, ^7Me^GTP, 3-deaza-adenosine, SIBA (5′-S-isobutylthio-5′-deoxyadenosine), sinefungin (adenosyl-ornithine), ribavirin (1-β-D-ribofuranosyl-1,2,4-triazole-3-carboxamide), MTA (5′-deoxy-5′-methylthio-adenosine), 2′,3′,5′-tri-O-acetyladenosine, S-5′-adenosyl-L-cysteine, 1,2-(((3-(4-methylpenyl)adamantine-1-yl)cabomoyl) and ATA (aurintricarboxylic acid); TriLink Biotechnologies: Ribavirin-triphosphate and ribavirin. EICAR-(5-Ethynyl-1-β-D-ribofuranosylimidazole-4-carboxamide)-triphosphate was a kind gift from P. Herdewijn (Leuven, Belgium). They were dissolved in H_2_O or DMSO as previously described [18], [52], [53] and ATA was dissolved in 0.1 M NaOH as described in [52]. Concentrations were set to 10 mM and compounds stored at -20°C. All radioactive reagents were purchased from Perkin Elmer.

### Cloning of the SARS-CoV nsp10, nsp14 and nsp16 genes

The SARS-CoV nsp10-, nsp14-, and nsp16-coding sequences were amplified by RT-PCR from the genome of SARS-CoV Frankfurt-1 (accession number AY291315) as previously described [72]. The nsp10, nsp14, and nsp16 genes (encoding residues 4231–4369, 5903–6429, and 6776–7073 of replicase pp1ab) were cloned using Gateway technology (Invitrogen) into expression vector pDest14 (pDest14/6His-nsp10, pDest14/6His-nsp14 and pDest14/6His-nsp16) to produce recombinant proteins carrying an N-terminal His_6_-tag. The nsp14 gene was also cloned into the pTXB1 vector from the Impact kit (New England Biolabs) to generate the pTXB1-nsp14 plasmid that allows the expression of the nsp14 protein fused to the intein-chitin binding domain.

SARS-CoV nsp10/nsp16 complex was produced in *E. coli* in a bi-promotor expression plasmid kindly provided by Bruno Coutard (AFMB France). In this backbone, SARS CoV nsp10 can be expressed under a tet promoter and encodes a protein in fusion with a N-terminal strep tag, whereas nsp16 is expressed under a T7 promoter and encodes a protein in fusion with a N-terminal hexa-histidine tag. The single point mutants of pDest14/6His-nsp14 (the mutant numbering starts at the beginning of the nsp14 sequence; D90A & E92A, H268A, H268L, D273A and D331A) and the mutants of pDest14/6His-nsp16 (the mutant numbering starts at the beginning of the nsp16 sequence; K46A, D130A, K170A, E203A) were generated by PCR using the Quickchange site–directed mutagenesis kit (Stratagene), according to the manufacturer's instructions.

### Expression and purification of SARS-CoV nsp10, nsp14 and nsp16 proteins

*E. coli* C41 (DE3) cells (Avidis SA, France), containing the pLysS plasmid (Novagen), were transformed with the various expression vectors and grown in 2YT medium containing ampicillin and chloramphenicol. Protein expression was induced by addition of IPTG to a final concentration of 500 µM (nsp10) or 50 µM (nsp14 and nsp16), when the OD_600 nm_ value of the culture reached 0.5. Nsp10 expression was performed during 4 h at 37°C, whereas nsp14- and nsp16-expressing bacteria were incubated during 16 h at 17°C. Bacterial cell pellets were frozen and resuspended in lysis buffer (50 mM HEPES, pH 7.5, 300 mM NaCl, 5 mM MgSO_4_, 5 mM β-mercaptoethanol (only for nsp10) supplemented with 1 mM PMSF, 40 mM imidazole, 10 µg/ml DNase I, and 0.5% Triton X-100. After sonication and clarification, proteins were purified by two steps of chromatography except the nsp14 mutants, which were purified by one-step of IMAC (HisPurTM Cobalt Resin; Thermo Scientific) and concentrated on 50-kDa centrifugal filter units (Millipore).

Two-step purification of the His_6_-tagged proteins started with IMAC (HisPurTM Cobalt Resin; Thermo Scientific) eluting with lysis buffer supplemented with 250 mM imidazole. Protein fractions were then loaded on a HiLoad 16/60 Superdex 200 gel filtration column (GE Healthcare), and eluted with 10 mM HEPES, pH 7.5, 150 mM NaCl. The protein fractions were concentrated to around 2 mg/ml and stored at −20°C in the presence of 50% glycerol.

The nsp14 protein expressed in fusion with the intein-chitin binding domain was purified on a chitin column using the IMPACT kit (New England Biolabs). The bacterial lysate was loaded onto the column, washed with 50 mM HEPES pH 7.5, 1 M NaCl and 0.5% Triton X-100. The column was then incubated in 50 mM HEPES pH 7.5, 500 mM NaCl, 50 mM DTT at 4°C for 48 hours in order to induce the intein cleavage. Next, the protein was eluted in 50 mM HEPES pH 7.5, 1 M NaCl buffer and subsequently purified on a HiLoad 16/60 Superdex 200 gel filtration column (GE Healthcare) as describe above. The identity of each of the purified proteins was confirmed by MALDI-TOF after trypsin digestion.

SARS-CoV nsp10/nsp16 co-expression was performed in *E. coli* strain C41 (DE3) (Avidis SA, France) transformed with the pLysS plasmid (Novagen). Cultures were grown at 37°C until the OD_600nm_ reached 0.6. Expression was induced by adding 50 µM IPTG and 200 µg/L of anhydrotetracycline; then cells were incubated for 16 h at 24°C. Bacterial pellets were treated as given above and the soluble protein fraction incubated with Strep-Tactin sepharose (IBA Biotagnology). After 3 washes, bound proteins were eluted with 2.5 mM D-desthiobiotin in binding buffer. After analysing the purified protein complex by SDS-PAGE, the intensities of Coomassie-stained bands were quantified using ImageJ.

### RNA synthesis and purification

Short capped RNAs (^7Me^GpppAC_5_, GpppAC_5_, were synthesized *in vitro* using bacteriophage T7 DNA primase and were purified by high-performance liquid chromatography (HPLC) as previously described [69].

RNA substrate corresponding to the 5′-terminal 264 nucleotides of the SARS-CoV genome (5′ SARS-264) was prepared as follows. The 5′ UTR of the SARS-CoV genome Frankfurt-1 was amplified by PCR using the primers BamH1-T7phi2.5-5′SARS (s) (CGGGATCCCAGTAATACGACTCACTATTATATTAGGTTTTTACCTACCC) and EcoRI-SARS-264 (as) (GGAATTCCTTACCTTTCGGTCACAC) and cloned in the pUC18 (Fermentas) plasmid after BamHI/EcoRI restriction-ligation procedure. The T7 class II Φ2.5 promoter [43] was used (underlined in the primer) and the second nucleotide of the genome (U) was substituted by a G. The transcription matrix, was amplified by PCR (primers BamH1-T7phi2.5-5′SARS-AG (s) (CGGGATCCCAGTAATACGACTCACTATTAGATTAGGTTTTTACCTACCC) and SARS-264 (as) (CTTACCTTTCGGTCACAC)) and purified on agarose gel using the QIAquick gel extraction kit (Qiagen). The AG-SARS-264 RNA substrate was synthesized by *in vitro* transcription using the MEGAshortscript T7 RNA polymerase kit (Ambion). After DNase treatment (Ambion), and purification by RNeasy mini kit (Qiagen), the AG-SARS-264 RNA was incubated for 1 h at 37°C with the VV capping enzyme (ScriptCap m7G Capping kit, Epicentre Biotechologies) in a reaction volume of 20 µl, either in the absence or in the presence of AdoMet, according to the instructions of the manufacturer. 10 µCi [α-^32^P]-GTP (PerkinElmer, Boston, MA) and 0.05 units of inorganic pyrophosphatase (Sigma–Aldrich) were used. Radiolabeled capped RNAs GpppAG-SARS-264 and ^7Me^GpppAG-SARS-264 were then purified with the RNeasy mini kit (Qiagen).

### Radioactive methyltransferase and filter binding assay

MTase activity assays were performed in 40 mM Tris-HCl, pH 8.0, 5 mM DTT, 1 mM MgCl_2_ (only for nsp16/nsp10), 2 µM ^7Me^GpppAC_5_ or GpppAC_5_, 10 µM AdoMet, cand 0.03 µCi/µl [^3^H]AdoMet (GE Healthcare). In the standard assay, nsp10, nsp14, and nsp16 were added at final concentrations of 1.2 µM, 50 nM, and 200 nM, respectively. The final concentrations of nsp14 and nsp16 used in the assays were chosen so as to stay in the linear phase of product formation after a 1 h incubation when using GpppAC_5_ or ^7Me^GpppAC_5_ as substrates. A 6-fold molar excess of nsp10 over nsp16 was chosen to achieve about 75% of the maximal stimulation of 2′O-MTase activity. Under these conditions, the nsp14 and nsp10/nsp16 methylation reactions converted similar amounts of substrate after 1 h of reaction. No sign of protein inactivation was found up to the apparent end of the linear phase. Reaction mixtures were incubated at 30°C and stopped after the indicated times by a 10-fold dilution of the reaction mixture in 100 µM ice-cold AdoHcy. Samples were kept on ice and then transferred to glass-fiber filtermats (DEAE filtermat; Wallac) by a Filtermat Harvester (Packard Instruments). Filtermats were washed twice with 0.01 M ammonium formate, pH 8.0, twice with water, and once with ethanol, dried, and transferred into sample bags. Betaplate Scint (Wallac) scintillation fluid was added, and the methylation of RNA substrates was measured in counts per minute (cpm) by using a Wallac 1450 MicroBeta TriLux liquid scintillation counter. For inhibition assays, we set up the reactions as described above with ^7Me^GpppAC_5_ for nsp16 and GpppAC_5_ for nsp14 in the presence of 100 µM inhibitor candidate. Enzymes and RNA substrates were mixed with the inhibitor before the addition of AdoMet to start the reaction. The final concentration of DMSO in the reaction mixtures was below 5%, and control reactions were performed in presence of DMSO, which does not alter MTase activity. Reaction mixtures were incubated at 30°C for 4 h and analyzed by filter binding assay as described above. The IC_50_ (inhibitor concentration at 50% activity) value of AdoHcy, sinefungin and ATA were determined using Kaleidagraph. Data were adjusted to a logistic dose-response function, % activity  = 100/(1+[I]/IC_50_)^b^, where b corresponds to the slope factor and [I] corresponds to the inhibitor concentration [73].

### MTase assay on SARS-264 RNA and cap-methylation analysis

MTase activity assays were performed in 40 mM Tris-HCl, pH 8.0, 5 mM DTT, 1 mM MgCl_2_ (only for nsp16/nsp10), 50 µM AdoMet and 0.75 µM of capped AG-SARS-264 RNA at 30°C. The reaction was stopped after different reaction times by incubating samples for 5 min at 70°C. Samples were treated overnight with proteinase K (0.1 µg/µl, Invitrogen). Proteinase K was inactivated by addition of 5 mM PMSF; and the RNAs were subsequently digested for 4 h with nuclease P1 (0.05 U/µl, USBiological). Radiolabeled cap analog standards were produced by direct digestion of the substrates leading to GpppA and ^7Me^GpppA or digestion after methylation of the 2′O-position using VV 2′O-MTase (ScriptCap 2′O-methyltransferase kit, Epicentre Biotechnologies) leading to GpppA_2'__OMe_ and ^7Me^GpppA_2'__OMe_. Digestion products were separated on polyethyleneimine cellulose thin-layer chromatography (TLC) plates (Macherey Nagel) using 0.45 M (NH_4_)_2_SO_4_ as mobile phase. After drying TLC plates, the caps released by nuclease P1 were visualized using a phosphorimager (Fluorescent Image Analyzer FLA3000 (Fuji)).

## Supporting Information

Text S1 Optimization of the nsp14 and nsp16/nsp10 MTase activities on small capped RNA substrates GpppAC_5_ and 7^Me^GpppAC_5_, respectively.
(0.03 MB DOC)

Figure S1 Optimization of the nsp14 and nsp16/nsp10 MTase activities on small capped RNA substrates. Nsp14 or nsp16/nsp10 were incubated with GpppAC5 (in grey) or ^7Me^GpppAC_5_ (in black), respectively. The methyl transfer to the RNA substrates was determined after 30 min (panels A, C to E) or 1 h (panel B) by filter-binding assay. Data represent mean values of three independent experiments. Panel **A**: The pH dependence of the nsp14 N7-MTase activity (50 mM) and the 2′O-MTase activity of nsp16/nsp10 (200 nM/1.2 µM) was determined in 50 mM Bis-Tris (pH 5 to 7.5) and Tris-HCl buffer (pH 7 to 10). Values at determined pH optima were arbitrarily set to 100%. Panel **B**: The effect of increasing nsp10 concentration was determined on nsp14 (50 nM) and nsp16 (200 nM) MTase activities in Tris-HCl, pH 8.0 containing 0 (nsp14) or 1 mM (nsp16/nsp10) MgCl_2_. Values at optimum nsp10/nsp16-MTase ratios were arbitrarily set to 100%. Data points represent the mean of two independent experiments. Panels **C, D and E**: Effect of increasing concentrations of MgCl_2_, MnCl_2_, ZnCl_2_ on the nsp14 N7-MTase (50 nM) and nsp16 2′O-MTase (200 nM) incubated with a 6-fold excess of nsp10. Values obtained in Tris-HCl, pH 8.0; 5 mM DTT without ions for nsp14 and with 1 mM MgCl_2_ for nsp16/nsp10 were arbitrarily set to 100%.
(0.46 MB EPS)
