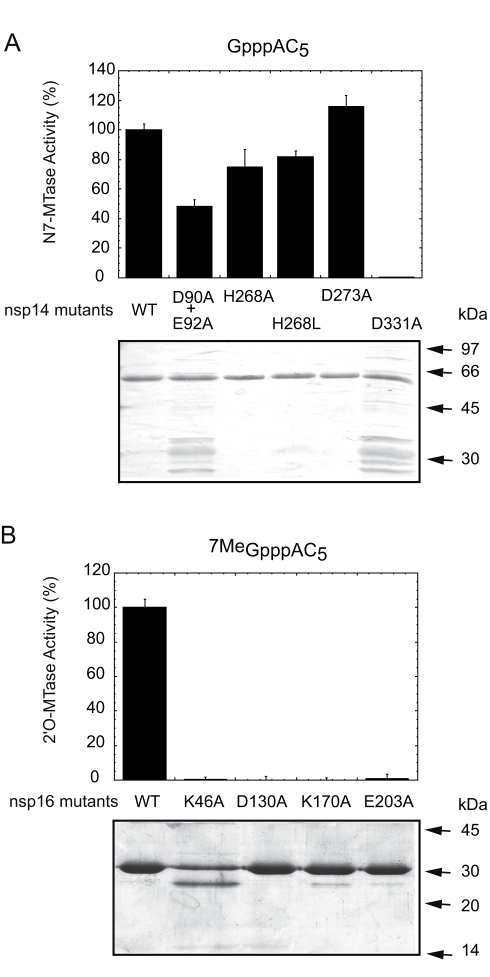# Correction: *In Vitro* Reconstitution of SARS-Coronavirus mRNA Cap Methylation

**DOI:** 10.1371/annotation/a0dde376-2eb1-4ce3-8887-d29f5ba6f162

**Published:** 2010-05-12

**Authors:** Mickaël Bouvet, Claire Debarnot, Isabelle Imbert, Barbara Selisko, Eric J. Snijder, Bruno Canard, Etienne Decroly

An error was introduced in the preparation of this article for publication. The incorrect figure file was provided for Figure 4. Please view the correct Figure 4 here: 

**Figure:**