# Supplementary material for: In Vitro Reconstitution of SARS-Coronavirus mRNA Cap Methylation
Source: PLoS Pathog. 2010 Apr 22;6(4):e1000863. doi: 10.1371/journal.ppat.1000863 (PMC2858705; doi:10.1371/journal.ppat.1000863)
Supplement: Text S1 — Optimization of the nsp14 and nsp16/nsp10 MTase activities on small capped RNA substrates GpppAC5 and 7MeGpppAC5, respectively. (0.03 MB DOC) [file ppat.1000863.s001.doc]

**Text S1. Optimization of the nsp14 and nsp16/nsp10 MTase activities on small capped RNA substrates GpppAC_5_ and ^7Me^GpppAC_5_, respectively**

The pH optima were determined to be between pH values 8.0 and 9.0 for the N7-MTase nsp14 and between 7.0 and 8.0 for the 2'O-MTase activity of nsp16/nsp10 (Figure S1A). Adding nsp10 at increasing concentrations to both MTase tests, we confirmed that nsp14 is weakly activated by nsp10 and that nsp16 is stimulated in a dose-dependent manner by nsp10 reaching a plateau of optimum activity at a 10-fold molar excess over nsp16 (Figure S1B). Furthermore, nsp14 was not activated by metal ions as Mg^2+^, Mn^2+^ and Zn^2+^ and inhibited in a progressive manner at concentrations higher than 1 mM MgCl_2_, 0.1 mM MnCl_2_ or 2.5 mM ZnCl_2_ (Figure S1C to S1E). In contrast, Mg^2+^ and Mn^2+^ were equally efficient as catalytic ions for the 2’O-MTase activity of nsp16/nsp10 with optima for MgCl_2_ between 0.5 and 5 mM and for MnCl_2_ between 0.5 and 1 mM (Figure S1C and S1D). Zn^2+^ ions were 10-fold less efficient as catalytic ions (Figure S1E). Finally, NaCl addition had a negative effect on both MTase activities at concentrations higher than 50 mM and the presence of reducing agent DTT (5 mM) stabilized the MTases during long time course experiments (data not shown).

**Figure** **S1.** **Optimization of the nsp14 and nsp16/nsp10 MTase activities on small capped RNA substrates**

Nsp14 or nsp16/nsp10 were incubated with GpppAC_5_ (in grey) or ^7Me^GpppAC_5_ (in black), respectively. The methyl transfer to the RNA substrates was determined after 30 min (panels A, C to E) or 1 h (panel B) by filter-binding assay. Data represent mean values of three independent experiments. Panel **A**: The pH dependence of the nsp14 N7-MTase activity (50 mM) and the 2’O-MTase activity of nsp16/nsp10 (200 nM/1.2 µM) was determined in 50 mM Bis-Tris (pH 5 to 7.5) and Tris-HCl buffer (pH 7 to 10). Values at determined pH optima were arbitrarily set to 100 %. Panel **B**: The effect of increasing nsp10 concentration was determined on nsp14 (50 nM) and nsp16 (200 nM) MTase activities in Tris-HCl, pH 8.0 containing 0 (nsp14) or 1 mM (nsp16/nsp10) MgCl_2_. Values at optimum nsp10/nsp16-MTase ratios were arbitrarily set to 100%. Data points represent the mean of two independent experiments. Panels **C, D and E**: Effect of increasing concentrations of MgCl_2_, MnCl_2_, ZnCl_2_ on the nsp14 N7-MTase (50 nM) and nsp16 2’O-MTase (200 nM) incubated with a 6-fold excess of nsp10. Values obtained in Tris-HCl, pH 8.0; 5 mM DTT without ions for nsp14 and with 1 mM MgCl_2_ for nsp16/nsp10 were arbitrarily set to 100%.
